# Impact of the COVID-19 Pandemic on Saudi Parents’ Perceptions Toward Their Children’s Oral Health: A Cross-Sectional Study

**DOI:** 10.7759/cureus.67125

**Published:** 2024-08-18

**Authors:** Rola M Alhossine, Randa M Ibrahim

**Affiliations:** 1 Department of General Dentistry, College of Dentistry, Qassim University, Qassim, SAU; 2 Department of Orthodontics and Pediatric Dentistry, College of Dentistry, Qassim University, Qassim, SAU

**Keywords:** fear, attitude, parents, oral health, children, covid-19

## Abstract

Background

The coronavirus disease 2019 (COVID-19) markedly affected all aspects of life. Pediatric dentistry and oral health of children faced a challenging situation during the pandemic.

Aim

This study aimed to assess the attitude of Saudi parents toward their children's oral health and dental treatment during the COVID-19 pandemic.

Methods

This online-based cross-sectional study was conducted using a structured questionnaire adopted from previous studies with some modifications. It was distributed among 385 Saudi parents recruited through social media. It included questions regarding demographic data, parents’ attitudes toward their children's dental care and dental visits during the COVID-19 pandemic, their fear level and willingness to take their children to dental appointments, and the parent’s perception of dental clinics with regard to the COVID-19 spread. Descriptive statistics and the chi-square test were used; statistical significance was set at a p-value < 0.05.

Results

Different levels of fear of the pandemic were expressed by Saudi parents, with 42 (10.9%) showing maximum fear while 36 (9.3%) reported no fear. Although about half of the parents stated that their children experienced dental pain during the pandemic, only 97 (25.1%) looked for dental care and were assisted. There was a significant association (p < 0.05) between the fear level with the family income and parents’ willingness to take their children to dental appointments.

Conclusions

COVID-19 has had a great impact on parents’ attitudes towards their children's oral health. A high percentage of Saudi parents' stated that they would take their children to dental clinics during the pandemic only for urgent treatment and severe pain.

## Introduction

On March 11th, 2020, the coronavirus disease 2019 (COVID-19) was declared a pandemic by the World Health Organization (WHO) [1]. The highly contagious virus spreads rapidly through respiratory droplets expelled by infected individuals or from contact transmission with contaminated objects [2]. Thus, dentistry practice and dental clinics are considered high-risk settings for viral transmission among patients and dental personnel [3,4].

To combat the spread of COVID-19, most governments, including the Kingdom of Saudi Arabia, set control measures such as lockdowns, curfews, social distancing, and quarantine. Although the incidence of COVID-19 in Saudi Arabia increased and decreased, the statistics showed remarkable control. This is attributed to the committed governmental efforts and the disciplined attitude of the public [5].

Suspension of schools and outdoor activities and increased time at home have led to alterations in the lifestyle and health behaviors of children [4-6]. Subsequently, changes in dietary habits and oral hygiene practices occurred, and access to dental care was greatly compromised [6-8]. Henceforth, pediatric dentistry faced a challenging situation that compromised the oral health of children [6,9-12].

Moreover, the concerns regarding the safety of both dental practitioners and patients have led to a decrease in routine professional dental care [12-14]. Fear of the pandemic has greatly influenced even the use of emergency dental services [15]. It is only natural that the concerns of parents regarding COVID-19 are remarkably high when it comes to their children. However, as parents play a crucial role in the oral health of their children, their perception of COVID-19 was reported by some authors to have a negative impact on their children’s oral health and oral care [13,16,17].

Thus, this study aimed to assess the attitude of Saudi parents toward their children's dental treatment and care during the COVID-19 pandemic, their fear level, their willingness to take their children to dental appointments, and their confidence level in the dental clinics after learning that the clinics had taken protective measures.

## Materials and methods

Ethical considerations

The Ethical Committee of the Dental Research Center at Qassim University (Saudi Arabia) approved this study (EA/F-2020-5003). All participants were requested to agree to the informed consent attached with the questionnaire before being included in the current study.

Study design

The study was conducted at College of Dentistry, Qassim University, Saudi Arabia. It is an online questionnaire-based cross-sectional study that was conducted from November 2020 to January 2021 during the second wave of the COVID-19 pandemic.

Sampling and study participants

The sample size was calculated, using a 95% confidence level and a margin of error of 5%, as a minimum of 385 respondents.

A convenient snowball-sampling technique was used to recruit participants via social media platforms of the Saudi population. Google Form link and invitations to participate in the study were distributed using the investigators profiles in WhatsApp (Meta Platforms, Inc., Menlo Park, California, United States), Twitter (Twitter, Inc., San Francisco, California, United States), Telegram (Telegram, Dubai, United Arab Emirates), and Instagram (Meta Platforms, Inc., Menlo Park, California, United States). The responding participants were then requested to spread invitations to their contacts.

Inclusion criteria were Saudi parents (fathers or mothers) of at least one child less than 12 years of age, parents of healthy children with no special health care needs, parents consenting to participate, and parents who had an account on social media. Parents of nationalities other than Saudi and those with special health care needs children were excluded.

Data collection

A self-administered structured questionnaire was designed based on previous studies [13,18] with some modifications. The questionnaire was translated from English to Arabic and then back translated to ensure accuracy. The pre-final Arabic version of the questionnaire was then pilot-tested to check its reliability among 38 parents (10% of the sample size) who were not included in the main study. The final version of the questionnaire is shown in Appendix 1.

After consenting, the participants answered 14 questions regarding socioeconomic and demographic data, including the parent’s age, relationship to the child (mother or father), number of children in the family, educational level of the parent, and family income. In addition, participants were asked to assess their fear level of the pandemic in an ascending order from 0 to 5. Other questions comprise parents’ perceptions towards their children’s oral health and dental visits during the COVID-19 pandemic and their confidence toward the protective measures taken by the dental clinics to reduce COVID-19 spread.

Statistical analysis

The statistical analysis was performed using the IBM SPSS Statistics for Windows, Version 26 (Released 2019; IBM Corp., Armonk, New York, United States). Descriptive statistics (frequencies, percentages, means, and standard deviations) were done for the different variables in the study. The chi-square test was used to assess the association among different categorical variables in the study. A p-value of less than 0.05 was considered to be statistically significant.

## Results

A total of 387 participants responded to the questionnaire; the majority were mothers (n=296) compared to 91 fathers. Demographic data on parents’ age and level of education, number of children, and family income are shown in Table 1.

**Table 1:** Demographic distribution of data. Data are presented in numbers and percentages (%).

Age (in years)	N (%)
Less than 20	31 (8.0)
20-29	97 (25.1)
30-40	147 (38.0)
More than 40	112 (28.9)
Relationship to child	N (%)
Mother	296 (76.5)
Father	91 (23.5)
Number of children in family	N (%)
One child	94 (24.3)
From 2 to 5	204 (52.7)
More than 5	89 (23.0)
Education	N (%)
Primary school or less	71 (18.3)
Intermediate or high school	133(34.4)
University or higher	183 (47.3)
Family income	N (%)
Low	47 (12.1)
Middle	271 (70.0)
High	69 (17.8)

Regarding the frequency of brushing their children's teeth during the pandemic, 162 (41.9%) parents reported brushing their children's teeth occasionally, 120 (31.0%) once per day, and only 105 (27.1%) brush twice or more per day.

The distribution frequency of dental trauma, the presence of caries lesions, and dental pain as reported by parents are shown in Table 2. Although about half of the parents (n=186, 48.1%) stated that their children experienced dental pain during the pandemic, only 97 parents (25.1%) looked for dental care and were assisted, 28 (7.2%) were not assisted, and 61 (15.8%) did not look for dental care. As for parents who reported their children having dental trauma, about half of them (n=69, 17.8%) stated looking for dental care and being assisted.

**Table 2:** Distribution of dental pain, presence of caries, and dental trauma as reported by parents. Data are presented in numbers and percentages (%).

	No	Yes, I looked for dental care and my child was assisted	Yes, I looked for dental care but we were not assisted	Yes, but I did not look for dental care
Pain	201 (51.9%)	97 (25.1%)	28 (7.2%	61 (15.8%)
Trauma	247 (63.8%)	69 (17.8%)	25 (6.5%)	46 (11.9%)
Caries	223 (57.6%)	64 (16.5%)	23 (5.9%)	77 (19.9%)

**Figure 1:**
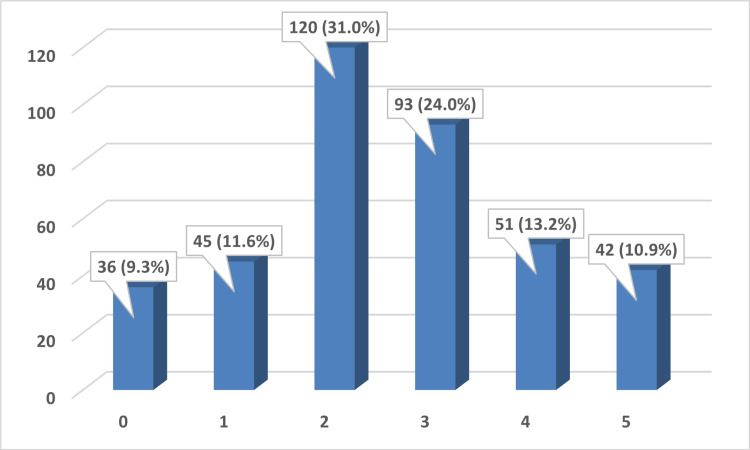
Fear level of the pandemic.

Figure 1 shows that most parents displayed intermediate levels of fear, with 120 (31%) and 93 (24%) reporting levels 2 and 3, respectively. On the other hand, the two extreme levels 0 and 5 were reported by only 36 (9.3%) and 42 (10.9%) consecutively.

**Figure 2:**
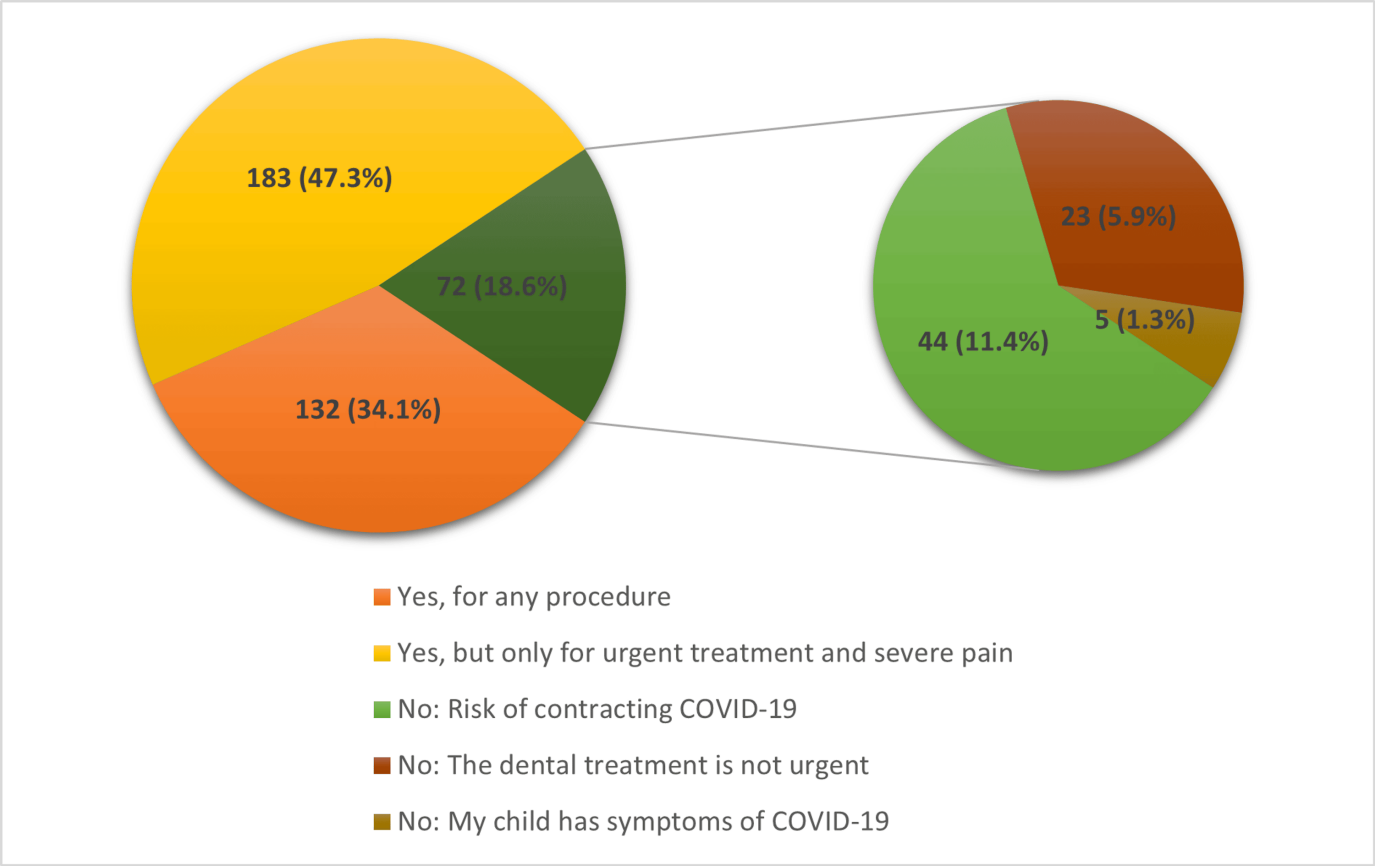
Parents' willingness to take their children to dental appointments during the pandemic.

Nearly half of the parents (n=183, 47.3%) stated that they would take their children to dental appointments only for urgent treatment and severe pain, while 72 (18.6%) would not take them at all. Among the latter group, the main reason, according to 44 (11.4%) parents, was fear of contracting the COVID-19 virus (Figure 2).

Table 3 illustrates the parents’ views for dental treatment and dental clinics with regard to COVID-19 spread. Only 106 (27.4%) parents thought that the environment of the dental departments was more dangerous than that of other public places. The majority of parents (n=337, 87.1%) reported having confidence in dental treatment after learning that dental clinics had taken protective measures.

**Table 3:** Parents’ perception of dental clinics with regard to the COVID-19 spread. Data are presented in numbers and percentages (%).

Parents’ beliefs if the environment of the dental clinics is more dangerous than that of other public places	N (%)
Yes	106 (27.4%)
Similar	143 (37.0%)
No	138 (35.7%)
Parents’ confidence in the dental clinics after learning that the clinics had taken protective measures	N (%)
Yes	337 (87.1%)
No	50 (12.9%)

There was no significant association between the fear level and any of the demographic data (parents’ age, their level of education, number of children in the family). However, a significant association (p<0.05) was noted between the fear level and the participating parent (father or mother), the family income, and parents’ willingness to take their children to dental appointments (Table 4).

**Table 4:** Association of parents’ fear level of COVID-19 with demographic variables and their willingness to take their children to dental appointments during the pandemic. χ2 test: chi-square test; * P-value > 0.05 is significant Data are presented in numbers and percentages (%).

	Fear level	0	1	2	3	4	5	χ^2 ^Test	P-value
Age of the parent (in years)	> 20	4 (1.0%)	1 (0.3%)	7 (1.8%)	10 (2.6%)	5 (1.3%)	4 (1.0%)	23.232	0.079
	20-29	12 (3.1%)	11 (2.8%)	27 (7.0%)	25 (6.5%)	17 (4.4%)	5 (1.3%)		
	30-40	7 (1.8%)	20 (5.2%)	57 (14.7%)	29 (7.5%)	13 (3.4%)	21 (5.4%)		
	< 40	13 (3.4%)	13 (3.4%)	29 (7.5%)	29 (7.5%)	16 (4.1%)	12 (3.1%)		
Parent	Mother	27 (7.0%)	28 (7.2%)	77 (19.9%)	80 (20.7%)	44 (11.4%)	40 (10.3%)	30.89	0.000*
	Father	9 (2.3%)	17 (4.4%)	43 (11.1%)	13 (3.4%)	7 (1.8%)	2 (0.5%)		
Number of children in the family	One child	11 (2.8%)	4 (1.0%)	32 (8.3%)	30 (7.8%)	10 (2.6%)	7 (1.8%)	16.459	0.087
	From 2 to 5	16 (4.1%)	28 (7.2%)	68 (17.6%)	44 (11.4%)	26 (6.7%)	22 (5.7%)		
	More than 5	9 (2.3%)	19 (4.9%)	20 (5.2%)	19 (4.9%)	15 (3.9%)	13 (3.4%)		
Level of education	Primary school or less	14 (3.6%)	10 (2.6%)	20 (5.2%)	14 (3.6%)	5 (1.3%)	8 (2.1%)	17.602	0.062
	Intermediate or high school	10 (2.6%)	18 (4.7%)	41 (10.6%)	34 (8.8%)	20 (5.2%)	10 (2.6%)		
	University or higher	12 (3.1%)	17 (4.4%)	59 (15.2%)	45 (11.6%)	26 (6.7%)	24 (6.2%)		
Family income	Low	5 (1.3%)	6 (1.6%)	10 (2.6%)	12 (3.1%)	5 (1.3%)	9 (2.3%)	31.176	0.01*
	Middle	18 (4.7%)	26 (6.7%)	97 (25.1%)	69 (25.5%)	40 (10.3%)	21 (5.4%)		
	High	13 (3.4%)	13 (3.4%)	13 (3.4%)	12 (3.1%)	6 (1.6%)	12 (3.1%)		
Parents’ willingness to take their children to dental appointments during the pandemic	Yes, for any procedure	18 (4.7%)	27 (7.0%)	41 (10.6%)	20 (5.2%)	17 (4.4%)	9 (23.6%)	61.820	0.000
	Yes, but only for urgent treatments and severe pain	14 (3.6%)	12 (3.1%)	67 (17.3%)	57 (14.7%)	21 (5.4%)	12 (3.1%)		
	No	4 (1.0%)	6 (1.6%)	12 (3.1%)	16 (4.1%)	13 (3.4%)	21 (5.4%)		

## Discussion

The spread and consequences of COVID-19 varied considerably across different countries, leading to subsequent variations in responses and attitudes of the communities. In Saudi Arabia, this was governed by factors such as population size, urban growth, infrastructure, and economic status. The spread of COVID-19 in Saudi Arabia had initially prompted panic responses in the society. However, the measures created by the public health system combined with good community compliance had provoked effective control of the pandemic [5]. 

Dental care was greatly compromised due to the high hazards of viral transmission in dental clinics. More than other times, the general and oral health of children depends largely on their parents’ beliefs and attitudes.

The results of the current study showed that many parents are limited looking for professional dental care for their children. Besides that, a considerable number of parents stated they were looking for dental care for varied reasons (pain, trauma, and caries) but were not assisted. The data obtained are broadly consistent with the trends of other studies [13,19-21]. On the contrary, in a study from China [18], the majority of parents (83.78%) stated they would take their children to dental clinics in cases of severe pain. Limiting or deferring the provision of professional pediatric dental care was an unavoidable precaution during the pandemic, in spite of its expected unfavorable consequences for immediate and future oral health [22].

Many reports and guidelines worldwide emphasize that attending a dental clinic poses an extra risk of COVID-19 transmission through aerosol generation during many dental procedures [23]. Surprisingly, different studies have shown apparent variations in the perceptions of participants regarding this issue. The findings of the current study state that just about a quarter of the participants (27.4% of parents) thought that dental clinics constitute more danger than other public places. Similar findings were reported in other studies from Saudi Arabia (36%) [24] and Turkey (34%) [25]. These rather low percentages might indicate insufficient awareness among the public about how the COVID-19 virus might be transmitted in dental clinics and accordingly highlight the need for more educational programs. On the contrary, other studies reported higher awareness; 53% of participating parents in India [26] and 66% in China [18] thought the dental environment could be more dangerous than other environments. However, the high confidence level about protective measures taken by dental clinics reported in the current study (87.1%) is consistent with another study in China (81%) [18]. Alternatively, other studies show lower levels of confidence. A study from Saudi Arabia [24] reported that more than half of participating parents had little or no confidence in the infection control measures taken at dental offices. Similarly, 46% of the respondents in an Indian study showed confidence in the preventive measures of dental departments [26].

In the current study, various levels of fear of the pandemic were expressed by the participants, with 10.9% showing maximum fear. These findings are consistent with prior studies that noted variable levels of fear. In a study in Brazil [13], 16.1% of participants declared high levels of fear; another study in India [27] reported that 64% had high fear levels. While this might seem normal, the high levels of fear might cause people to have unreasonable thoughts regarding many issues; their children’s oral health is no exception. A significant association between the fear level and the parent’s willingness to take their children to dental clinics was noted in the current study; about half of the parents with the highest level of fear (score 5) would not take their children to dentists. This was consistent with other studies from Brazil [13], India [16], Italy [28], and Saudi Arabia [24]. These results reflect that the COVID-19 pandemic might lead to an increase in unmet treatment needs and thus negatively affect the oral health of children. Limited research has compared the pre- and post-dental status of children; a study from Greece [29] revealed more carious lesions and significantly increased treatment needs after the lockdown.

The present study highlights important clues about the favorable and unfavorable attitudes of parents regarding their children's oral health during the disturbed period of the pandemic and thus provides a path to improve educational programs. Further implementation of alternative tools, such as teledentistry, might be useful in the future. This can be achieved by remote parents' education regarding not just general oral hygiene. It is also necessary to put more emphasis on topics such as home care, infection control, and modes of transmission of the COVID-19 virus. Furthermore, the public needs education about dental emergencies and the importance of professional intervention in such cases while maintaining strict protection protocols.

The current study has some limitations, such as the relatively small sample size. Furthermore, the data collected via an online questionnaire might be a potential bias since the findings might not be representative of a sector of parents with no social media accounts who might stand for certain educational or socioeconomic levels. Another limitation is the subjectivity of some of the questions, such as the socioeconomic status and dental caries being noticed by parents. However, a community-based sampling technique would not have been possible because of the social distancing and lockdown measures during the study period.

## Conclusions

This study provides additional evidence that COVID-19 had a significant impact on parents attitudes towards their children’s oral health. About half of the Saudi parents in the current study stated that they would take their children to dental clinics during the pandemic, only for urgent treatment and severe pain. A considerable number of parents expressed fear of the pandemic; this was significantly associated with family income and parents’ willingness to take their children to dental appointments. Most of the parents showed confidence in the protective measures taken by the dental clinics. The findings might imply that further future work should be done on oral health education and infection control. Further research is needed to elaborate on the provision of pediatric dental treatment during the pandemic and to evaluate the changes in the oral health of children and related attitudes and awareness pre- and post-COVID-19.

## Appendices

Appendix 1

Questionnaire

Impact of the COVID-19 Pandemic on Saudi Parents’ Perceptions Toward Their Children’s Oral Health

I would appreciate your assistance with this research project about the impact of the COVID-19 pandemic on Saudi parents perceptions toward their children’s oral health.

If you are a Saudi parent (father or mother) for at least one child less than 12 years of age and your child is healthy with no special health care needs, then please proceed.

All you need to do is complete this short questionnaire, which should take approximately three minutes. Responses will be completely anonymous; your name will not appear anywhere on the survey. Completing and returning the questionnaire constitutes your consent to participate.

1- How old are you (in years):

1.     More than 20

2.     20-29

3.     30-40

4.     Less than 40

2- Relationship to child:

1.     Mother

2.     Father

3- Number of children in family:

1.     1

2.     2-5

3.     More than 5

4- What is your highest level of education completed:

1.     Primary school or less

2.     Intermediate or high school

3.     University or higher

5- Family income:

1.     Low

2.     Middle

3.     High

6- Were you able to regularly brush your children's teeth during the pandemic:

1.     Yes

2.     No

3.     Sometimes

7- Did any of your children experience dental trauma during the pandemic:     

1.     No

2.     Yes, I looked for dental care right after the trauma and my child was assisted

3.     Yes, I looked for dental care, but we were not assisted

4.     Yes, but I did not look for dental care

8- Did you notice any cavities/caries in your children's teeth during the pandemic:

1.     No

2.     Yes, I looked for dental care and my child was assisted

3.     Yes, I looked for dental care, but we were not assisted

4.     Yes, but I did not look for care

9- Did any of your children experience dental pain during the pandemic:     

1.     No

2.     Yes, I looked for dental care and my child was assisted

3.     Yes, I looked for dental care but we were not assisted

4.     Yes, but I did not look for dental care

10- On a scale of 0 to 5, where 0 is no fear and 5 is maximum fear, indicate the option that best describes your fear of the pandemic:

1.     0

2.     1

3.     2

4.     3

5.     4

6.     5

11- Would you take your child to a dental appointment during the pandemic:     

1.     Yes, for any procedure

2.     Yes, but only for urgent treatments and severe pain

3.     No

12- If not, for what reason:   

1.     Risk of contracting COVID-19

2.     The dental treatment is not urgent

3.     My child/I has/have symptoms of COVID-19.

13- Do you think the environment of the dental department is more dangerous than that of other public places:

1.     Yes

2.     Similar

3.     No

14- The dental department has taken various protective measures according to the requirements of the health committee, including patient screening, hospital environment disinfection, and special protective equipment for both dentists and patients. Will these measures give you confidence in dental treatment:

1.     Yes

2.     No
